# Examining health apps and wearable use in improving physical and mental well-being across U.S., China, and Singapore

**DOI:** 10.1038/s41598-024-61268-z

**Published:** 2024-05-11

**Authors:** Edmund W. J. Lee, Huanyu Bao, Yongda S. Wu, Man Ping Wang, Yi Jie Wong, K. Viswanath

**Affiliations:** 1 Wee Kim Wee School of Communication and Information, Nanyang Technological University, Singapore, Singapore; 2 Children’s Hospital of Eastern Ontario Research Institute, Ottawa, Canada; 3 School of Nursing, The University of Hong Kong, Hong Kong, Hong Kong SAR; 4 Dana-Farber Cancer Institute, Boston, USA; 5 Department of Social and Behavioral Sciences, Harvard T. H. Chan School of Public Health, Harvard University, Boston, USA

**Keywords:** Health apps, Wearable technology, Physical health, Mental well-being, Individualist and collectivist cultures, Social determinants of health, Health policy, Health services, Public health

## Abstract

Health apps and wearables are touted to improve physical health and mental well-being. However, it is unclear from existing research the extent to which these health technologies are efficacious in improving physical and mental well-being at a population level, particularly for the underserved groups from the perspective of health equity and social determinants. Also, it is unclear if the relationship between health apps and wearables use and physical and mental well-being differs across individualistic, collectivistic, and a mix of individual–collectivistic cultures. A large-scale online survey was conducted in the U.S. (individualist culture), China (collectivist culture), and Singapore (mix of individual–collectivist culture) using quota sampling after obtaining ethical approval from the Institutional Review Board (IRB-2021-262) of Nanyang Technological University (NTU), Singapore. There was a total of 1004 respondents from the U.S., 1072 from China, and 1017 from Singapore. Data were analyzed using multiple regression and negative binomial regression. The study found that income consistently had the strongest relationship with physical and mental well-being measures in all three countries, while the use of health apps and wearables only had a moderate association with psychological well-being only in the US. Health apps and wearables were associated with the number of times people spent exercising and some mental health outcomes in China and Singapore, but they were only positively associated with psychological well-being in the US. The study emphasizes the importance of considering the social determinants, social–cultural context of the population, and the facilitating conditions for the effective use of digital health technologies. The study suggests that the combined use of both health apps and wearables is most strongly associated with better physical and mental health, though this association is less pronounced when individuals use only apps or wearables.

## Introduction

Mobile health apps and wearables are touted to have the potential to revolutionize health interventions by increasing motivation for health behaviors uptake among the general population, as well as transform healthcare delivery by enhancing patient-provider care and access. In recent years, advances and breakthroughs in the deployment of health apps and wearable technologies in the health space have increased their credibility as a form of digital intervention, for example, as digital therapeutic treatment for opioid use disorder alongside existing clinical treatment methods^1^. Apps have also been useful in supporting clinicians in remote monitoring of patients’ health outcomes, adherence to medication and other recommended behaviors^2,3^.

Beyond innovations within the healthcare space, there is increasing attention to how health apps and wearables could improve physical and mental health in the general population. From a population health perspective, health apps and wearables are widely used for promotion of physical activity and healthy lifestyle^4^, inform tobacco intervention efforts^5^ and enhance behavioral and mental health outcomes or assess depressive symptoms^6,7^. For these “upstream” health interventions, the “big” data captured and generated by health apps and wearables can be rich in providing insights into consumers and patients’ behavior within their contextual environment. Depending on their features, apps and wearables could come with in-built sensors that capture data such as geocoordinates and mobility patterns, Bluetooth data for estimating proximity to social contacts, or physiological information pertaining to sleep, physical activity, electrodermal and cardiac activity^8^. As these data are often collected in a passive and unobtrusive “naturally occurring setting”, the moment-by-moment quantification of physiological, behavioral, and contextual data and make for a comprehensive digital exhaust for prediction of a variety of diseases^9^.

While there is much promise in the use of health apps and wearables in improving various facets of physical and mental health, researchers have documented that the implementation of digital health technologies often suffers from a major barrier known as *data absenteeism*^10^. Data absenteeism is the phenomenon where in a data-rich world, information and data about how health technologies improve underserved groups’ (across racial, ethnic, gender, resources, and geography) health are largely missing, or in some instances systematically excluded, despite the capacity of these technologies to generate critical health data at-scale and speed^11^. This is a critical component of social determinants and communication inequalities, where underserved populations are less likely to be able to take advantage of the benefits of digital innovations because of issues surrounding access, capacity, and information processing^12^. For instance, underserved populations are least likely to enroll and participate in randomized controlled trials or digital health interventions^13,14^, which impedes the use of big data and machine learning algorithms in understanding the causes of disparities and how best to use evidence to address them^15^.

Data absenteeism may manifest in two ways when it comes to apps and wearables. First, one of the current research gaps on data absenteeism is that it is unclear how health apps and wearables would improve physical and mental well-being at the population-level, given that results from research are mixed at best^16^. After all, the typical users of health apps and wearables are of a specific niche profile—the young, well-educated, and financially well-resourced^17^. In other words, a large proportion of the population, particularly underserved populations, may not actually be benefitting from health apps and wearables despite the ubiquity of smartphone ownership. It is not surprising that many of the commercial apps and wearables are developed by profit-driven companies that are targeting people with resources as their consumer base. Even when individuals in low-resourced settings have access to health apps and wearables, many may not be able to take advantage of these gadgets to their fullest potential due to issues surrounding *technology maintenance*—the on-going costs required for the upkeep (e.g., paying for internet connectivity, data plans) in order to continuously use these technologies effectively^18^.

Second, there is a lack of data on how *culture* could play a role in influencing how populations accept and interact with certain technologies^19^, and the COVID-19 pandemic has made some of these cultural differences more prominent. In the U.S., scepticism towards the government, scientific institutions, and technology companies is not uncommon, a sentiment rooted in historical distrust and concerns over privacy and autonomy^11^. This may contrast with attitudes observed in China and Singapore, where there is a collective acceptance of technology as a means to enhance societal well-being. For example, in China, the collective nature of society may predispose individuals to perceive technology as a communal good, thus facilitating its adoption^20^. Similarly, in Singapore, the blending of individualistic and collectivistic cultural traits creates a unique context where technology is widely embraced, even among older adults who are traditionally considered affected by the digital divide^21^. These differences may highlight the interplay between cultural norms and technology acceptance, which suggests that the effectiveness of health apps and wearables is also influenced by the cultural context in which they are deployed.

Given the issue of data absenteeism in health apps and wearables use in population health, the aims of this research are twofold. First, it examines the extent to which health apps and wearables could promote physical health (i.e., the amount of time people spent exercising and self-reported health) and mental well-being (i.e., psychological, emotional, and social) by partially addressing the issue of data absenteeism through sufficient inclusion of lower income and education groups. Second, it aims to compare if there are differences in how health apps and wearables relate to physical health and mental well-being across three different cultures: individualist (i.e., U.S.), collectivist (i.e., China), and a mix of both individualistic and collectivistic cultures (i.e., Singapore).

## Method

### Procedure and sample

We conducted a large-scale online survey in the U.S., China, and Singapore between October 2021 and January 2022. We adopted a quota sampling strategy, aligning our sample demographics with the national profiles of each country regarding gender, age, education, and ethnicity. This approach was informed by the latest census data available from the U.S., China, and Singapore. Participation in the survey was restricted to citizens and permanent residents aged 21 and above in all three countries. This age threshold was particularly relevant for Singapore, where the legal age for providing consent without parental approval is 21. The survey took about 20 minutes to complete and was administered in English in the U.S. and Singapore, and in Mandarin in China. There was a total of 1004 respondents from the U.S., 1072 from China, and 1017 from Singapore.

### Ethics committee approval

This research obtained ethics approval from the Nanyang Technological University, Singapore’s Institutional Review Board (IRB-2021-262). This study was conducted in accordance with all relevant guidelines and regulations for national surveys in China, Singapore, and the U.S. All procedures, including the collection, storage, and analysis of data, complied with the ethical standards of the relevant national data protection laws and institutional requirements. Prior to participation in the survey, informed consent was obtained from all participants. The study was carried out in accordance with the principles of the Declaration of Helsinki.

## Measurements

### Use of health apps and wearables

#### Use of health apps and wearables

To measure participants’ use of health apps and wearables, they were asked to indicate if they had used or were currently using a list of health mobile applications or wearables (e.g., Apple Health, WeChat Run, Apple Watch). We provided participants with a list of the top ten most frequently downloaded Apple and Android apps, as well as the top ten most commonly used health wearables, in the U.S., China, and Singapore. While the main focus of the study was not to determine the degree of comparability of health apps and wearables across participants from the three countries, to ensure consistency we generated a list of apps and wearables based on their popularity (e.g., iOS and Android market rankings for health apps) and the features related to physical activity, mental health support, and meditation, which would relate to the main health outcomes (exercise frequency, self-reported general health, and mental well-being) of the study. We have also selected apps and wearables that were globally available (e.g., Fitbit, Apple Watch), but also included apps and wearables that were only available in the specific countries. For instance, apps such as “Woman&Child HealthPedia” and 美柚" (Meiyou) which cater to women and child health are available only in Singapore and China respectively. To be exhaustive in capturing the types of health apps and wearables, participants were given the option to list other apps and wearables they used if they did not appear in the survey. The data were then dummy coded (0 = No; 1 = Yes) into three variables—*health apps use only*, *wearables use only*, and *health apps and wearables use*, where the reference group was those who neither used health apps nor wearables.

#### Frequency of health apps and wearable use

A single self-report item asked participants to estimate the frequency of their health app and/or wearable usage (in minutes per day) through the following statement: “In the past week, on average, approximately how many minutes per day have you spent using health wearables/apps?”.

### Physical health and mental well-being

#### Self-reported general health and number of times exercised

The self-reported general health of participants was self-rated through a single item, “Overall, how would you rate your health during the past 4 weeks?”, using a 5-point Likert scale (1 = “*Poor”* to 5 = *“Excellent”*). Participants were also asked to self-report their physical activity through the following statement: “During the past few weeks … how many times per week did you participate in any physical activities or exercises such as running, calisthenics, golf, gardening, or walking for exercise?”.

#### Mental well-being

Participants were asked questions pertaining to three different dimensions of mental well-being: psychological, emotional, and social well-being adapted from Lamers et al.^22^. The 14 items for all three mental well-being dimensions were measured on a 5-point Likert scale to assess the extent to which participants agreed with the statements (1 = *“Strongly disagree”* to 5 = *“Strongly agree”*). Examples of questions measuring each dimension include: “During the past month, how often did you feel happy” (emotional well-being); “During the past month, how often did you feel that you had something important to contribute to society” (psychological well-being); and “During the past month, how often did you feel: - that you had warm and trusting relationships with others” (social well-being). All scales had good internal reliability with high Cronbach’s α in the U.S. (psychological well-being: Cronbach’s α = 0.87; emotional well-being: Cronbach’s α = 0.90; social well-being: Cronbach’s α = 0.87), China (psychological well-being: Cronbach’s α = 0.88; emotional well-being: Cronbach’s α = 0.88; social well-being: Cronbach’s α = 0.90), and Singapore (psychological well-being: Cronbach’s α = 0.92; emotional well-being: Cronbach’s α  = 0.92; social well-being: Cronbach’s α = 0.93).

### Data analysis

To understand the relationship between social determinants, apps and wearables use with general health and the three mental health outcomes, we used ordinary least squares (OLS) regression. Independent variables included social determinants—gender, age, income, education, ethnicity, and urbanicity—as well as variables related to *health app use only*, *wearable use only*, *health and wearables use*, and *time spent on health apps and wearables*. To analyze the results for predicting the number of times exercised in a week, we fitted a negative binomial regression because the dependent variable (number of times exercised) exhibited overdispersion and contained a high number of zero values.

## Results

The U.S., Chinese, and Singaporean participants’ demographic information, including age, gender, ethnicity, income, education, and residential area, is comprehensively displayed in Table 1. Table 2 shows the specific apps and wearables used by respondents in the three countries, while Tables 3, 4, and 5 show the relationship between demographic factors and technology use and health outcomes.

**Table 1:** Demographic characteristics of samples from the US, China and Singapore.

Demographic characteristic	Number of respondents		
	US (N = 1004)	CN (N = 1072)	SG (N = 1017)
Gender			
Male	462 (46.00%)	545 (50.80%)	528 (51.90%)
Female	540 (53.80%)	521 (48.60%)	489 (48.10%)
Non-binary	2 (.20%)	6 (.60%)	0 (.00%)
Age			
Mean age (SD)	*49.91 (17.36)*	*38.26 (11.85)*	*42.49 (12.87)*
21–34	234 (23.30%)	492 (45.90%)	318 (31.30%)
35–54	348 (34.66%)	462 (43.10%)	507 (49.80%)
≥ 55 years	422 (42.03%)	118 (11.00%)	192 (18.90%)
Ethnicity (US)			
White American	644 (64.10%)	/	/
Hispanic	134 (13.30%)	/	/
Black	140 (13.90%)	/	/
Asian	65 (6.50%)	/	/
Others	21 (2.10%)	/	/
Ethnicity (CN)			
Han	/	1021 (95.20%)	**/**
Zhang	/	25 (2.30%)	/
Hui	/	17 (1.60%)	/
Others	/	9 (.80%)	/
Ethnicity (SG)			
Chinese	/	/	830 (81.60%)
Malay	/	/	95 (9.30%)
Indian	/	/	69 (6.80%)
Eurasian	/	/	3 (.30%)
Others	/	/	19 (1.90%)
Unknown	/	/	1 (.10%)
Average Annual Household Income (US)			
$0 to $49,999	452 (45.00%)	/	/
$50,000 to $99,999	316 (31.50%)	/	/
$100,000 to $149,999	144 (14.30%)	/	/
$150,000 to $199,999	55 (5.50%)	/	/
$200,000 or more	37 (3.70%)	/	/
Average Monthly Household Income (CN)			
Less than $1000 to $9999	/	587 (54.80%)	/
S$10,000 to S$19,999	/	292 (27.20%)	/
S$20,000 and above	/	193 (18.00%)	/
Average Monthly Household Income (SG)			
Less than $1000 to $9999	/	**/**	654 (64.30%)
S$10,000 to S$19,999	/	**/**	307 (30.20%)
S$20,000 and above	/	**/**	56 (5.50%)
Education (US)			
Less than 8 years	9 (.90%)	**/**	/
8 through 11 years	24 (2.40%)	**/**	/
12 years or completed high school	176 (17.50%)	**/**	/
Post high school training other than college (vocational or technical)	41 (4.10%)	**/**	/
Some college	220 (21.90%)	**/**	/
College graduate	339 (33.80%)	**/**	/
Postgraduate	193 (19.20%)	**/**	/
Others	2 (.20%)	**/**	/
Education (CN)			
No formal education	/	0 (.00%)	/
Primary school	/	5 (.50%)	/
Junior school	/	29 (2.70%)	/
Secondary vocational school	/	5 (.50%)	/
High school	/	118 (11.00%)	/
College degree	/	219 (20.40%)	/
University degree	/	580 (54.10%)	/
Master's degree	/	88 (8.20%)	/
PhD	/	28 (2.60%)	/
Others	/	0 (.00%)	/
Education (SG)			
No formal education	/	/	0 (.00%)
Primary 6 or below	/	/	4 (.40%)
Some secondary	/	/	16 (1.60%)
N-level/ITE	/	**/**	39 (3.80%)
O-level	/	/	39 (3.80%)
A-level/Diploma	/	/	142 (14.00%)
Degree	/	/	599 (58.90%)
Postgraduate	/	/	177 (17.40%)
Others	/	/	1 (.10%)
Urbanicity			
Remote	17 (1.70%)	23 (2.10%)	/
Rural	234 (23.30%)	96 (9.00%)	/
Suburban	410 (40.80%)	219 (20.40%)	/
Urban	340 (33.90%)	734 (68.50%)	/
Prefer not to say	3 (.30%)	0 (.00%)	/

**Table 2:** Types of health apps and wearable used by respondents from the U.S., China, and Singapore.

Types of health apps and wearables	Number of respondents		
	US (N = 1004)	CN (N = 1072)	SG (N = 1017)
Health wearables used			
Apple Watch	271 (26.99%)	303 (28.26%)	206 (20.26%)
Samsung Smartwatch	174 (17.33%)	85 (7.93%)	137 (13.47%)
Huawei Watch Fit	68 (6.77%)	369 (34.42%)	58 (5.70%)
Fitbit Watch (e.g., Fitbit Sense, Fitbit Versa)	148 (14.74%)	58 (5.41%)	155 (15.24%)
Fitbit Tracker (e.g., Fitbit Inspire, Fitbit Charge)	152 (15.14%)	60 (5.60%)	177 (17.40%)
Mi Smart Band	55 (5.48%)	321 (29.94%)	51 (5.01%)
Huawei Band	33 (3.29%)	240 (22.39%)	30 (2.95%)
Other _______	26 (2.59%)	7 (0.65%)	100 (9.83%)
I never use it	474 (47.21%)	262 (24.44%)	361 (35.50%)
Health apps used			
Apple Health	262 (26.10%)	286 (26.68%)	236 (23.21%)
Samsung Health	173 (17.23%)	85 (7.93%)	210 (20.65%)
Huawei Health	66 (6.57%)	454 (42.35%)	60 (5.90%)
Fitbit: Health & Fitness	159 (15.84%)	**/**	/
Mi Fit	52 (5.18%)	268 (25.00%)	67 (6.59%)
Planet Fitness Workouts	48 (4.78%)	**/**	/
My FitnessPal	82 (8.17%)	**/**	/
Flo Ovulation Period Tracker	37 (3.69%)	**/**	/
Calm	62 (6.18%)	**/**	/
Motivation-Daily quotes	37 (3.69%)	**/**	/
Replika-My AI Friend	23 (2.29%)	**/**	/
All Trails: Hike, Bike&Run	19 (1.89%)	**/**	/
Keep	/	209 (19.50%)	/
美柚	/	93 (8.68%)	/
薄荷健康	/	59 (5.50%)	/
每日瑜伽	/	88 (8.21%)	/
小睡眠	/	62 (5.78%)	/
丁香妈妈	/	37 (3.45%)	/
HealthHub SG	/	**/**	205 (20.16%)
Healthy 365 app	/	**/**	480 (47.20%)
ActiveSG App	/	**/**	114 (11.21%)
AICare Link App	/	**/**	14 (1.38%)
Mobile E-care Locator (MEL)	/	**/**	16 (1.57%)
KneeBuddy App	/	**/**	8 (0.79%)
MyEyeGym	/	**/**	10 (0.98%)
Health Buddy Appf	/	**/**	65 (6.39%)
Woman&Child HealthPedia	/	**/**	11 (1.08%)
NUH myMeds	/	**/**	19 (1.87%)
Other _______	32 (3.19%)	8 (0.75%)	32 (3.15%)
I never use it	443 (44.12%)	164 (15.30%)	164 (16.13%)

**Table 3:** Outcomes of health apps and wearables use in the U.S.

	Estimates for exercise frequency	Estimates for general health	Estimates for psychological well-being	Estimates for emotional well-being	Estimates for social well-being
Social determinants					
Gender (Reference = Male)					
Female	− 0.05	− 0.03	− 0.09*	− 0.04	0.03
Binary	0.00	0.04	− 0.02	0.00	0.02
Age	− 0.09*	− 0.02	0.11*	0.31***	0.28***
Income	0.06	0.21***	0.18***	0.25***	0.21***
Education	− 0.02	0.03	0.06	0.04	0.03
Ethnicity (Reference = White)					
Black	− 0.01	0.02	0.02	0.05	0.05
Hispanic	0.03	0.04	0.08	0.04	0.04
Asian	0.04	− 0.02	− 0.02	− 0.03	− 0.04
Others	0.03	0.00	− 0.03	0.02	0.01
Urbanicity (Reference = Urban)					
Suburban	− 0.07	− 0.07*	− 0.15***	− 0.10*	− 0.07
Rural	− 0.05	− 0.10**	− 0.11*	− 0.01	− 0.04
Apps & wearables use					
Apps only	− 0.02	0.00	0.03	0.01	0.03
Wearables only	− 0.02	− 0.01	0.01	− 0.02	− 0.03
Apps & wearables	0.05	0.05	0.23***	0.09	0.09
Time spent on apps & wearables (minutes)	0.06	0.02	− 0.03	− 0.03	0.02

**Table 4:** Outcomes of health apps and wearables use in China.

	Estimates for times exercised	Estimates for general health	Estimates for psychological well-being	Estimates for emotional well-being	Estimates for social well-being
Social determinants					
Gender (Reference = Male)					
Female	0.00	0.02	0.07*	0.06	0.08*
Age	0.04*	− 0.08**	0.04	0.05	0.05
Income	0.04	0.24***	0.20***	0.22***	0.19***
Education	0.00	− 0.05	− 0.03	− 0.02	− 0.01
Ethnicity (Reference = Han)					
Zhuang	− 0.03	− 0.03	− 0.02	− 0.01	0.01
Hui	0.02	0.00	0.00	0.04	− 0.02
Others	− 0.03	0.01	0.03	0.02	0.02
Urbanicity (Reference = Urban)					
Suburban	− 0.02	− 0.06*	− 0.03	− 0.30	− 0.02
Rural	0.00	− 0.02	0.00	− 0.02	− 0.04
Apps & wearables use					
Apps only	0.07**	0.05	0.07	0.06	0.09*
Wearables only	0.00	0.00	0.00	0.00	0.00
Apps & wearables	0.14***	0.08	0.19***	0.14**	0.19***
Time spent on apps & wearables (minutes)	0.01	0.09**	0.08*	0.07*	0.05

**Table 5:** Outcomes of health apps and wearables use in Singapore.

	Estimates for times exercised	Estimates for general health	Estimates for psychological well-being	Estimates for emotional well-being	Estimates for social well-being
Social determinants					
Gender (Reference = Male)					
Female	− 0.05*	− 0.02	0.00	0.07	0.04
Age	0.06**	− 0.06*	0.18***	0.25***	0.23***
Income	0.03	0.11***	0.18***	0.17***	0.18***
Education	0.08***	0.03	0.06	− 0.04	− 0.03
Ethnicity (Reference = Chinese)					
Malay	0.08***	0.14***	0.22***	0.25***	0.27***
Indian	0.11***	0.12***	0.24***	0.22***	0.20***
Eurasian	− 0.01	0.08**	0.07	0.07	0.08*
Others	0.00	0.03	0.06	0.01	0.03
Apps & wearables use					
Apps only	0.02	0.00	− 0.06	− 0.03	− 0.04
Wearables only	0.03	− .001	− 0.05	− 0.03	0.01
Apps & wearables	0.13***	0.10*	0.12*	0.06	0.08
Time spent on apps & wearables (minutes)	0.13***	0.07*	0.08	0.12**	0.13**

### Demographic information of respondents from the U.S., China, and Singapore

In the U.S. sample, the distribution of genders included 462 males (46%), 540 females (53.8%), and 2 individuals identifying as non-binary (0.2%). The Chinese cohort comprised of 545 males (50.8%), 521 females (48.6%), and 6 non-binary individuals (0.6%). In Singapore, the gender distribution was 528 males (51.9%) and 489 females (48.1%). Age-wise, the U.S. respondents spanned from 21 to 66 years, with an average age of 49.91 years (SD = 17.36). The Chinese participants' ages ranged from 21 to 63 years, with a mean age of 38.26 years (SD = 11.85), while the Singaporean sample's ages varied from 21 to 64 years, with a mean age of 42.49 years (SD = 12.87). Ethnic composition varied significantly across the samples. In the U.S., the majority were White (664 respondents, 64.1%), followed by Black (140 respondents, 13.9%), Hispanic (134 respondents, 13.3%), Asian (65 respondents, 6.5%), and Other (21 respondents, 2.1%). The Chinese sample was predominantly Han Chinese (1021 respondents, 95.2%), with smaller representations from Zhuang (25 respondents, 2.3%), Hui (17 respondents, 1.6%), and other ethnicities (9 respondents, 0.8%). The Singaporean respondents were mostly Chinese (830 respondents, 81.6%), with Malay (95 respondents, 9.3%), Indian (69 respondents, 6.8%), Asian-European (3 respondents, 0.3%), and other or unknown ethnicities (20 respondents, 2%) also represented.

### Outcomes of health apps and wearables use in the U.S.

For exercise frequency, there was a negative association between age and exercise frequency (β = − 0.09, *p* < 0.05), suggesting that older individuals tend to exercise less. For self-reported health, income had a positive association with it (β = 0.21, *p* < 0.001), indicating that higher-income individuals report better health outcomes. Compared to urban environment, residents of suburban (β = − 0.07, *p* < 0.05) and rural (β = − 0.10, *p* < 0.01) areas reported poorer self-reported general health, this points to possible disparities in healthcare access or environmental factors influencing health.

For psychological well-being, females had poorer outcomes (β = − 0.09, *p* < 0.05) compared to males. Younger individuals reported better psychological well-being (β = 0.11, *p* < 0.05), and higher income was also linked to better psychological health (β = 0.18, *p* < 0.001). Similar to self-reported general health, residents of suburban (β = − 0.15, *p* < 0.001) and rural (β = − 0.11, *p* < 0.05) areas reported lower psychological well-being than urban dwellers. Notably, users of health apps and wearables reported significantly better psychological well-being (β = 0.23, *p* < 0.001), suggesting the potential of digital health tools to support mental health.

For emotional well-being, the positive associations with age (β = 0.31, *p* < 0.001) and income (β = 0.25, *p* < 0.001) indicate that older and wealthier individuals perceive their emotional health more favorably. However, suburban residents reported slightly poorer emotional well-being (β = − 0.10, *p* < 0.05).

Lastly, both age (β = 0.28, *p* < 0.001) and income (β = 0.21, *p* < 0.001) showed significant positive associations with social well-being, highlighting the importance of these demographics in fostering a sense of social connectedness and support.

### Outcomes of health apps and wearables use in China

Contrary to trends observed in the U.S., there was a positive association between age and exercise frequency (β = 0.04, *p* < 0.05), which suggests a slight increase in physical activity with age. Furthermore, the use of apps alone (β = 0.07, *p* < 0.01) and in combination with wearables (β = 0.14, *p* < 0.001) was significantly associated with higher exercise frequency, highlighting the potential of digital health interventions to encourage physical activity.

For self-reported general health, a negative association with age (β = − 0.08, *p* < 0.01) indicates that older individuals may perceive their health more poorly, reflecting concerns about aging and health in China's rapidly aging society. Higher income (β = 0.24, *p* < 0.001) correlated with better self-reported health, underscoring socioeconomic status as a critical determinant of health perceptions. Suburban residents reported slightly poorer health outcomes (β = − 0.06, *p* < 0.05), similar to trends observed in the U.S., suggesting an urban–rural divide in health perceptions that warrants further investigation. The positive correlation between time spent on apps and wearables and general health (β = 0.09, *p* < 0.01) further supports the role of technology in enhancing health awareness and management.

Psychological well-being in China also showed significant associations with gender, with females reporting better outcomes (β = 0.07, *p* < 0.05), contrasting with findings from the U.S. This could reflect cultural differences in gender roles or social support systems. Additionally, higher income (β = 0.20, *p* < 0.001), the use of health apps and wearables (β = 0.19, *p* < 0.001), and time spent on health apps and wearables (β = 0.08, *p <* 0.05) were linked to better psychological well-being, emphasizing the importance of economic factors and digital health tools in mental health.

Emotional well-being was positively influenced by income (β = 0.22, *p* < 0.001) and the use of apps and wearables (β = 0.14, *p* < 0.01), suggesting that financial security and engagement with health technology contribute to positive emotional states. Time spent on these technologies (β = 0.07, *p* < 0.05) also had a beneficial effect, pointing to the value of digital engagement in emotional health.

Social well-being had significant associations with gender (β = 0.08, *p* < 0.05) and income (β = 0.19, *p* < 0.001). The use of apps alone (β = 0.09, *p* < 0.05) and in combination with wearables (β = 0.19, *p* < 0.001) had a positive impact on social well-being, underscoring the multifaceted role of digital tools in fostering social connections and support networks.

### Outcomes of health apps and wearables use in Singapore

For exercise frequency, females are less likely to engage in physical activities (β = − 0.05, *p* < 0.05). Age (β = 0.06, *p* < 0.01) and higher levels of education (β = 0.08, *p* < 0.001) positively correlate with increased exercise frequency, indicating that older and more educated individuals may have a greater awareness of the benefits of physical activity. Compared to Chinese, Malay and Indian ethnicities were associated with higher exercise rates (β = 0.08, *p* < 0.001; β = 0.11, *p* < 0.001, respectively), pointing to cultural or community-based factors that might encourage physical activity among these groups. Furthermore, the use of apps and wearables (β = 0.13, *p* < 0.001) and the time spent on these technologies (β = 0.13, *p* < 0.001) significantly enhanced exercise frequency, highlighting the potential of digital interventions in promoting physical health.

For self-reported general health, age exhibited a negative association (β = − 0.06, *p* < 0.05), suggesting that perceived health status may decline with advancing age. Conversely, higher income levels (β = 0.11, *p* < 0.001) are linked to better self-reported health outcomes, emphasizing the impact of socioeconomic status. Ethnicity also plays a significant role, with Malay (β = 0.14, *p* < 0.001), Indian (β = 0.12, *p* < 0.001), and Eurasian (β = 0.08, p < 0.01)  participants reporting better general health, which could reflect distinct cultural or lifestyle factors. The engagement with health apps and wearables (β = 0.10, *p* < 0.05) and the duration of their use (β = 0.07, *p* < 0.05) were positively associated with general health perceptions, underscoring the value of technology in health self-assessment and management.

Psychological well-being in Singapore was positively influenced by several factors, including age (β = 0.18, *p* < 0.001) and income (β = 0.18, *p* < 0.001), indicating that older and wealthier individuals tend to report higher levels of psychological well-being. Ethnic differences were also evident, with Malay (β = 0.22, *p* < 0.001) and Indian (β = 0.24, *p* < 0.001) participants reporting greater psychological health, possibly due to strong community support systems or cultural resilience. Additionally, the use of apps and wearables (β = 0.12, *p* < 0.05) contributed to better psychological well-being, highlighting the effectiveness of digital health tools in supporting mental health.

Emotional well-being was similarly associated with age (β = 0.25, *p* < 0.001) and income (β = 0.17, *p* < 0.001), with older and wealthier respondents experiencing better emotional health. The relationship between ethnicity and emotional well-being, particularly among Malay (β = 0.25, *p* < 0.001) and Indian (β = 0.22, *p* < 0.001) individuals, along with the use of apps and wearables (β = 0.12, *p* < 0.01), suggest that cultural factors and digital engagement were crucial to cultivating emotional well-being.

For social well-being, age (β = 0.23, *p* < 0.001), income (β = 0.18, *p* < 0.001), and ethnic background, including Malay (β = 0.27, *p* < 0.001), Indian (β = 0.20, *p* < 0.001), and Eurasian (β = 0.08, *p* < 0.05), show positive associations, indicating the significance of these demographic factors in fostering a sense of community and belonging. The engagement with technology, as seen in the use of apps and wearables (β = 0.13, *p* < 0.01), was positively associated with social well-being, reinforcing the role of digital tools in connecting and supporting individuals socially.

## Discussion

There are several key findings from this study pertaining to examining health apps and wearables use in the context of data absenteeism. First, when comparing the association between social determinants and health apps and wearables use with physical and mental health outcomes, income emerged consistently as the strongest antecedent for all three countries, suggesting that digital health technologies have limited effects at the population level. Second, in the U.S., apps and wearable use were largely ineffective in improving both physical and mental well-being, it only was positively associated with psychological well-being. This was in contrast with China and Singapore, where apps and wearables use were associated with the number of times people spent exercising, and some of the mental health outcomes. Third, we found that for all three countries, apps and wearables use were most effective in improving physical and mental health when used together.

First, while it is important to highlight the advances in digital interventions using health apps and wearables^1^, it is equally important for us to acknowledge that the relationship between health apps and wearables use and physical and mental well-being are perhaps minute, compared to traditional measures of social determinants. In the context of our study, specifically income level consistently had the strongest relationship with measures of physical health (number of times exercised and self-reported general health) as well as psychological, emotional and social well-being. In contrast, the relationship between the use of health apps and wearables and psychological well-being was moderate. While urbanicity (whether individuals lived in urban, suburban, or rural areas) were associated with physical and mental health outcomes in the U.S., this was not so in China and Singapore.. In terms of racial and ethnic measures, they mattered only in Singapore, and not in the U.S. or China. In Singapore, the minority races indicated better physical and mental health outcomes as compared to the reference category of Chinese, which constitutes about 75% of the population.

The data showed that it is pertinent for public health scholars, medical professionals, and technology developers to be keenly aware of the social–cultural context of the populations they are developing digital health technologies for, in order to avoid the problem of technology chauvinism. For instance, Lee et al. highlighted that any e-health interventions ought to account for and address the varying degrees of communication inequalities that populations are struggling with in their technology development plan^23^. This would mean developing digital interventions using health apps and wearables where people of all varying levels of income would have access to. In addition, researchers would need to pay attention to problems beyond “accessibility” such as the second-level of digital divide, which would include connectivity problems, as well as understanding how people take advantage of and use such technologies^24^.

The second most significant finding of our study showed that the impact of health apps and wearables is not unanimous, as there are cultural and country-specific differences in how they relate to physical and mental health. Surprisingly, in the U.S., the use of health apps and wearables was only positively associated with psychological well-being—whether one used only apps, or only wearables had no significant relationship with all outcomes. This is significant considering that a vast amount of health apps and wearables research is coming from western-centric samples. This suggests that at least in the U.S., the positive effects of health apps and wearables are largely psychological, and social determinants played more of an important role in explaining whether individuals are healthier or not, both physically and in terms of mental health.

In the U.S., the individualistic culture prioritizes personal autonomy and skepticism towards collective institutions, which may explain the limited motivational capacity of health technologies beyond psychological well-being enhancements. This cultural backdrop might influence the degree to which individuals are willing to integrate technology into their health routines, emphasizing the necessity for digital health interventions to align with values of privacy, autonomy, and personalization to enhance their acceptance and effectiveness. This problem is compounded by high-profile privacy and data violations such as unwanted tracking of sexual activity by Fitbit^25^, and that data from these devices are not protected like health information under the federal Health Insurance Portability and Accountability Act (HIPAA), further amplifying erosion of trust^26^.

In our Chinese sample, the use of health apps and wearables was positively associated with the number of times people exercised, as well as psychological, emotional, and social well-being, but not self-reported health. In other words, while health apps and wearables do motivate Chinese individuals  to exercise, which also improved their well-being, they do not necessarily translate to improvements in self-reported general health. This finding is consistent with existing research on China’s culture and technology acceptance. According to Hofstede’s cultural dimension, China ranks low in terms of individualism (as it is a more collectivistic society) and high in power-distance relationship with authorities^27^. Yang et al., in their study on the acceptance of wearable technology among Swiss and Chinese consumers, found that for the Chinese sample, social influence had a greater impact on behavioral intention to use wearables compared to the Swiss; the study showed that the Chinese sample showed a higher level of behavioral intention to use wearables compared to their Swiss counterparts^28^.

This is in contrast with Singapore, where the use of health apps and wearables was positively associated with the number of times people exercised, as well as self-reported health and psychological well-being. The government plays an active and pivotal role in setting the public health agenda and coordinating nation-wide digital health programs for preventive health, such as the National Steps Challenge, where Singaporeans are encouraged to walk 10,000 steps daily and they are given free wearable tracking devices (with a dedicated health app called Healthy 365) and monetary incentives for their involvement^29^. This appeals to both the collectivistic (emphasizing the norms of using technology for health) and individualistic (e.g., monetary incentives and health benefits) traits of the nation.

Third, we found that the combined use of both health apps and wearables had the strongest impact on physical and mental health, but this effect was less pronounced when individuals used only apps or wearables. This is consistent with research on technology acceptance, which often found that facilitating conditions—the conditions and infrastructure that make technology easy to use—are strong predictors of usage and adoption of any technology system^30^. After all, many of the existing health apps and wearables developed by mass market commercial companies are designed to be used collectively. While individuals could turn to “free” health apps, it is likely that many of these apps would not have the full suite of functions made available to them, and they may have their user-experience interrupted due to in-app advertisements.

Like all studies, there are some limitations in our study. First, we are cognizant that our attempts at ensuring sufficient representation of underprivileged groups to address the problem of data absenteeism does not comprehensively explore the extent to which broader systemic inequalities prevent these communities from achieving equitable health outcomes, despite the adoption of digital health technologies. While our study specifically examined the roles of some aspects of social determinants (i.e., income, education, ethnicity, distinction between rural and urban environment) and their relationship with health outcomes (compared to use of apps and wearables), there is a need to contextualize risk factors by understanding the underlying social conditions that are fundamental causes of certain diseases^31^. Link and Phelan^31^ argued that one reason why there is a consistent relationship between structural constraints and disease onset is the access to resources that may help an individual prevent or minimize health risks. One of such structural constraints would be the nature of *communication infrastructure* (information and interpersonal channels where individuals received health information) in different communities, where underprivileged groups may be disadvantaged in (a) getting accurate health information and (b) engaging in sense-making and taking advantage of these information for their health benefits^32^, as compared to their well-off counterparts. Individuals from underserved populations may also have a lack of diversity in their social networks compared to those with more resources^33^ and thus lack both interpersonal and institutional support in utilizing digital health technologies.

For future research in data absenteeism, we recommend researchers to undertake an in-depth investigation into the surrounding causes of data absenteeism in the context of digital health technologies use. This may involve complementing quantitative studies such as this with a more qualitative approach (through ethnographic studies or in-depth interviews), and examining the social and structural context of how individuals in underserved communities use digital health technologies. This would allow researchers to identify barriers to implementation from a policy perspective, as well as individual-level factors related to the adoption of digital health^33^.

Beyond our attempts at addressing data absenteeism, another limitation is that administering the survey over the Internet might attract participants from a particular demographic profile, particularly the higher income and more educated groups. However, we have oversampled the bottom income and education groups by 20% to ensure that the low SES groups were sufficiently represented. Finally, we acknowledge that people may use multiple apps and wearables for different purposes and future scholars should examine how motivations and usage of different apps may be associated with health outcomes.

## Conclusion

While recent breakthroughs in the use of health apps and wearables in promoting physical and mental health are much celebrated, it is important to be keenly aware of the issues surrounding data absenteeism and technology chauvinism. In other words, medical professionals, public health scholars and practitioners, and technology developers need to be mindful not to overstate the promises of health apps and wearables, considering the minute impact they have on physical and mental health at the population level across different countries. Therefore, when designing or deploying digital interventions, researchers and public health organizations must create technology that takes into account the social determinants and socio-cultural contexts of the populations they serve, while moving beyond merely providing sophisticated technical solutions or interfaces. By tackling data absenteeism and technology chauvinism from the get-go, it will ensure a more equitable design and implementation of health apps and wearables for different populations.

## Data Availability

Data will be made available on request, please contact Edmund Lee via edmundlee@ntu.edu.sg.
